# Supplementary figures and images for: Multiplex PCR Based Strategy for Detection of Fungal Pathogen DNA in Patients with Suspected Invasive Fungal Infections
Source: J Fungi (Basel). 2020 Nov 23;6(4):308. doi: 10.3390/jof6040308 (PMC7712097; doi:10.3390/jof6040308)

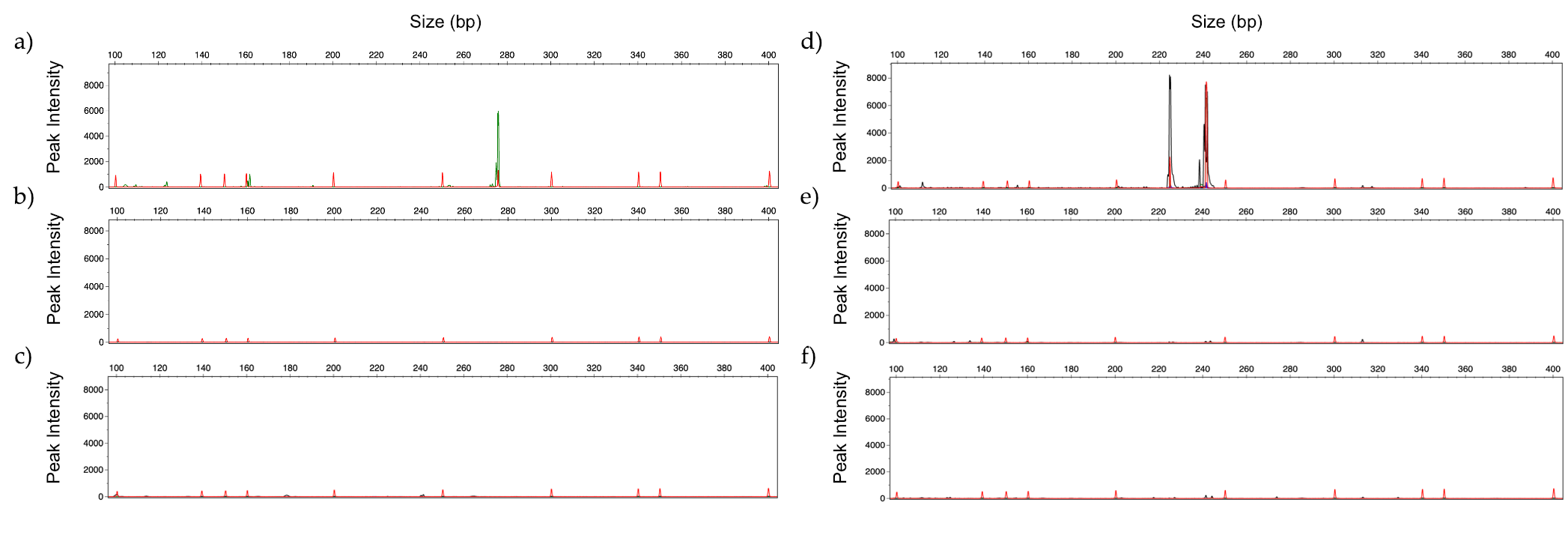

Supplement: Supplementary file 1 [file jof-06-00308-s001.zip › Figure S1.tif]

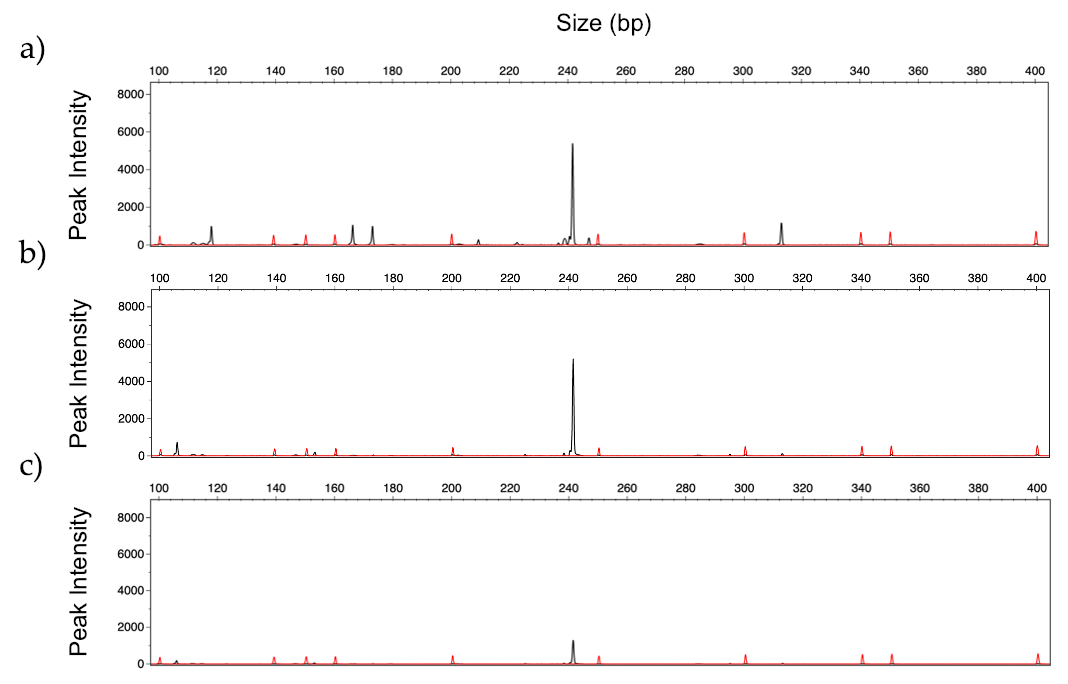

Supplement: Supplementary file 1 [file jof-06-00308-s001.zip › Figure S2.tif]

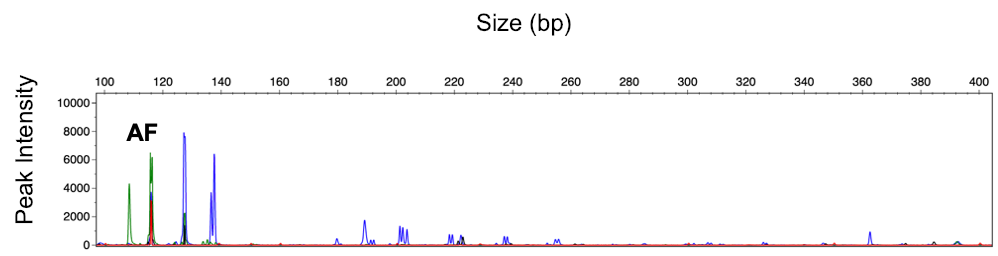

Supplement: Supplementary file 1 [file jof-06-00308-s001.zip › Figure S3.tif]
